# From Hematoxylin and Eosin to Masson’s Trichrome: A Comprehensive Framework for Virtual Stain Transformation in Chronic Liver Disease Diagnosis

**DOI:** 10.3390/diagnostics16050764

**Published:** 2026-03-04

**Authors:** Hossam Magdy Balaha, Khadiga M. Ali, Ali Mahmoud, Ahmed Aboudessouki, Mohamed T. Azam, Guruprasad A. Giridharan, Dibson Gondim, Ayman El-Baz

**Affiliations:** 1 Bioengineering Department, J.B. Speed School of Engineering, University of Louisville, Louisville, KY 40292, USA; hmbala01@louisville.edu (H.M.B.);; 2 Pathology Department, Faculty of Medicine, Mansoura University, Mansoura 35516, Egypt; 3 Department of Pathology and Laboratory Medicine, University of Louisville, Louisville, KY 40292, USA

**Keywords:** deep learning, stain normalization, stain conversion, histopathological analysis, transformers

## Abstract

**Background/Objectives**: Virtual histological staining offers a rapid, cost-effective alternative to physical reprocessing but faces challenges related to spatial misalignment and staining heterogeneity between Hematoxylin and Eosin (H&E) and Masson’s Trichrome (MT) domains. This study develops a robust framework for H&E-to-MT virtual staining to enable accurate fibrosis assessment without additional tissue consumption. **Methods**: We propose a transformer-based generative adversarial network (TbGAN) supported by a multi-stage alignment pipeline (SIFT (scale-invariant feature transform) coarse alignment, ORB/homography patch registration, and B-spline free-form deformation) and a weighted fusion mechanism combining four configuration outputs (O/10/3, O/3/10, R/10/3, and R/3/10). The framework was validated on 27 whole-slide images (>100,000 aligned patches) through 24 independent experiments. **Results**: The fused approach achieved state-of-the-art performance: MI = 0.9815 ± 0.0934, SSIM = 0.7474 ± 0.0597, NCC = 0.9320 ± 0.0220, and CS = 0.9946 ± 0.0014. Statistical analysis confirmed enhanced stability through narrower interquartile ranges, fewer outliers, and tighter 95% confidence intervals compared to individual configurations. Qualitative assessment demonstrated preserved collagen morphology critical for fibrosis staging. **Conclusions**: Our framework provides a reliable, IRB-compliant solution for virtual MT staining that maintains high structural fidelity suitable for diagnostic support. It enables resource-efficient fibrosis quantification and supports integration into clinical digital pathology workflows without patient-specific recalibration.

## 1. Introduction

Histopathological analysis is a primary tool of modern medicine, delivering vital data that helps in the recognition, categorization, and therapy of different diseases [1,2]. Liver fibrosis determination is one of the typical uses of histopathology as liver fibrosis is a common co-morbidity of chronic liver diseases such as non-alcoholic steatohepatitis (NASH), hepatitis B and C, and alcoholic liver cirrhosis [3,4]. Fibrosis (which is characterized by the excessive accumulation of extracellular matrix components such as collagen) is, therefore, not only a mainstay of the recognition of the progression of the condition but also a major factor in determining patient outcomes and therapeutic strategies [5,6].

Fibrosis assessment by pathologists is typically done through selective staining, which is a technique that focuses on specific tissue structures. The primary two stains are Hematoxylin and Eosin (H&E) and Masson’s Trichrome (MT) [7]. H&E staining is considered the best method for a regular histopathological examination, offering a detailed view of the cellular structure [8,9]. Hematoxylin binds to nuclei and gives them a blue-purple color; meanwhile, Eosin colors cytoplasmic and extracellular components pink. Thus, the experts can look at cellular organization and also, by comparing it to a normal one, can they easily determine the presence of inflammation, necrosis, or tumor cells or any other types of disease [10,11]. At the same time, in standard histological samples, H&E staining cannot easily reveal the differences between collagen and other connective tissues, the latter being the main source of fibrosis [12].

On the other hand, MT staining is a method which is aimed at giving new insights into collagen and various extracellular matrix components [13]. Here, collagen fibers in the samples are not only separated but also made visually prominent in blue color, and cells like hepatocytes and inflammatory cells are stained red. Due to this color differentiation, MT staining has become the main method in not only quantifying fibrosis but also, and most importantly, grading fibrosis severity [7,8]. For instance, when it comes to liver biopsies, MT staining is the most commonly used method to evaluate METAVIR (or Ishak) scoring systems as these systems differentiate the fibrosis stages by the extent and distribution of collagen accumulation [14,15,16].

However, the implementation of MT staining in clinical settings is limited by a number of practical challenges. To begin with, it requires extra tissue sections that may not be available routinely, especially in cases of biopsy specimens that are very small [17]. Secondly, the MT staining process is more costly and labor-intensive than H&E staining, limiting its feasibility in resource-constrained clinical settings [18]. Thirdly, MT and H&E slides come from different tissue cross-sections; thus, misalignments/inconsistencies and discrepancies in the spatial distribution of tissue components may occur. This makes it difficult to directly compare H&E and MT slides for accurate fibrosis determination.

In order to surmount these issues, the most recent attempts have been directed toward virtual staining, a computational approach that digitally alters images from one staining method to another [19]. In particular, the capacity to virtually transform H&E slides into MT ones may lead to many practical applications in the field of digital pathology. For example, in cases where only H&E slides are obtainable, virtual MT pathologists would be able to access fibrosis without the need for additional MT staining [20]. This can facilitate the diagnosis and treatment of chronic liver diseases, which need fibrosis evaluation as a basis for disease progression and drug regimens [21].

### 1.1. Advancements in H&E-to-MT Translation

Naglah et al. (in 2022) published a paper outlining the potential of conditional GANs (cGANs) for H&E-to-MT translation [22]. They reported a promising quantitative and qualitative performance in the generation of virtual MT slides from H&E WSIs that supported liver fibrosis detection and quantification without the demand for physical MT specimens [22]. Nonetheless, their method had difficulties that included: (a) the ceaseless mismatch of the H&E and MT slides, (b) variability in staining procedures; and (c) challenge of keeping the detailed collagen structures in the synthesized MT images, as follows:

(i) Local vs. global context modeling: cGANs operate through convolutional filters with limited receptive fields, capturing only local pixel neighborhoods. This impedes preservation of long-range collagen fiber continuity essential for fibrosis staging. Our suggestion is to utilize a transformer-based architecture that explicitly models global dependencies through self-attention mechanisms spanning the entire 256 × 256 patch.
(ii) Alignment dependency: Naglah et al. [22] required near-perfect pre-alignment of H&E/MT pairs, failing under common misalignments (>5% area mismatch). Our suggestion is to implement a multi-stage pre-processing pipeline (e.g., SIFT (scale-invariant feature transform) → ORB/homography → free-form deformation (FFD)) to report sub-pixel registration even with 15%:20% initial misalignment.
(iii) Stain variability robustness: cGANs exhibited significant performance degradation under staining protocol variations (SSIM drop: 0.18:0.23). Our suggestion is to think about a fusion mechanism that maintains stable performance across diverse staining conditions through complementary configuration aggregation.

### 1.2. Suggested Approach

Therefore, we propose a new virtual H&E-to-MT staining framework that significantly extends and goes beyond the work of Naglah et al. [22]. Our proposed method employs a transformer-based generative adversarial network (TbGAN), which combines the transformer’s global feature extraction capabilities with the GAN’s image generation power. While the cGAN-based method is a pixel-level transformation that changes the image locally, our TbGAN architecture is able to capture long-range dependencies in histopathological images; thus, it can generate MT slides that are not only more accurate but also more realistic. Furthermore, we have developed a full-fledged pre-processing pipeline comprising tile extraction, alignment, and FFD to rectify the misalignment problems pointed out in the previous work. This work, in essence, makes three major contributions:

(i) We put forward a TbGAN-based model architecture for H&E-to-MT stain transformation that outperforms the cGAN-based method by considering global context and producing more plausible collagen fibril structures.
(ii) We built a strong pre-processing pipeline that guarantees precise spatial matching of H&E and MT images, thus solving the alignment problems that were taken as a challenge in the previous work.
(iii) We proved the power of our method through a fair number of experiments, leading to drastic improvements in image similarity metrics and fibrosis detection accuracy as compared to the cutting-edge cGAN-based technique.

Table 1 quantifies the architectural and performance differentials between the suggested approach and Naglah et al.’s cGAN approach [22]. Critically, our suggested design eliminates the need for paired training data per institution, a key barrier to clinical deployment that plagues supervised cGAN approaches requiring site-specific retraining.

Collectively, our framework has the potential to transform digital pathology workflows, reducing reliance on physical MT staining, accelerating diagnostic turnaround, and enhancing the precision of fibrosis assessment in chronic liver disease and other fibrotic disorders. The remainder of the study is organized as follows: Section 2 details the study materials and proposed approach. Section 3 presents the experimental setup, results, and a comprehensive discussion of the study. Section 4 details the study limitations and discusses potential directions for future work. Finally, Section 5 concludes the paper.

## 2. Materials and Methods

**Figure 1:**
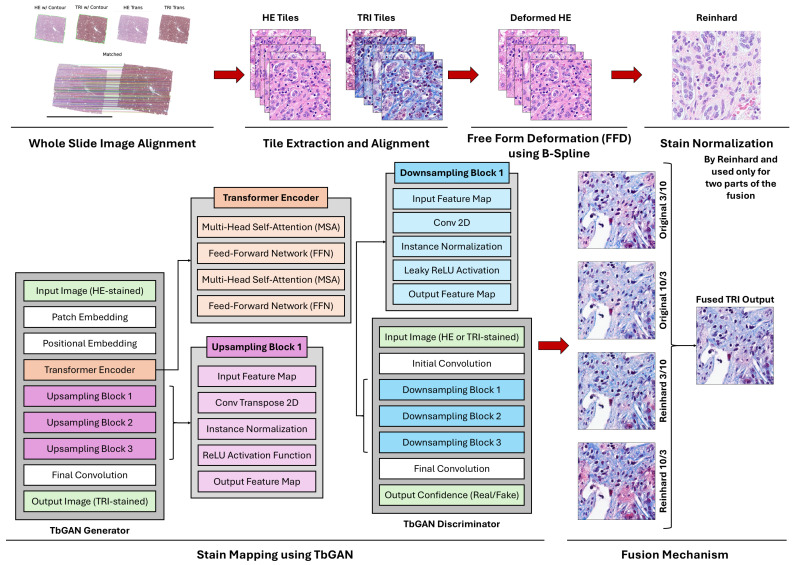
The proposed framework for virtual H&E-to-MT staining consisting of four main stages: (1) data pre-processing (including tile extraction and alignment); (2) stain normalization (using Reinhard’s method); (3) stain translation (using the proposed TbGAN); and (4) weighted fusion of multiple configurations (to enhance robustness and generalizability).

The proposed framework for H&E-to-MT virtual staining (refer to Figure 1) aims at helping fibrosis detection and quantification in histopathology (which has been traditionally left under-served) by using state-of-the-art deep learning (DL) techniques. A transformer-based generative adversarial network (TbGAN) along with a pre-processing pipeline is proposed in the work to digitally convert the H&E-stained images into virtual MT-stained ones in order to (i) support the fibrosis evaluation and diagnostic workflow enhancement; (ii) save material and time by cutting down on the physical re-staining; and (iii) open up possibilities of large-scale histopathological data analyses. Our framework’s features can be represented as four layers, where each of which has been thoughtfully designed to conquer the limitations of traditional staining methods:

(i) Data pre-processing: a robust pipeline for tile extraction, alignment, deformation, and normalization to ensure high-quality input data.
(ii) Stain Normalization: application of Reinhard’s normalization technique to standardize the color appearance/variability across images.
(iii) Transformer-based GAN (TbGAN): a proposed architecture merging the global feature extraction capabilities of transformers with the high-quality image generation/synthesis of GANs.
(iv) Fusion Mechanism: a weighted fusion approach to merge the synthetic outputs of multiple configurations, balancing identity preservation and cycle-consistency.

In the following subsections, we provide a detailed description of each step in the proposed framework, including data acquisition, pre-processing, stain normalization, and the architecture of the transformer-based GAN. We also discuss the fusion mechanism and its role in enhancing the robustness and generalizability of the framework.

### 2.1. Data Acquisition, Challenges, and Solution

The dataset for this research comprised pathologic whole slide images (WSIs) of liver tissue samples taken during liver transplantation surgeries from 27 human subjects/patients (27 for HE and 27 for MT; later processed to >100 K filtered tiles). The study secured the necessary approvals from the Institutional Review Board (IRB: 22.0448) and followed research ethics procedures for the University of Louisville (UofL).

Data Preparation Protocol: The liver tissue specimens were first fixed in the histopathology laboratory with paraffin and cut into 4-micron thin sections, after which the sections were placed on glass slides and stained with H&E. At the same time, adjacent sections were also placed on other glass slides and stained with MT. The H&E- and MT-stained slides were then both scanned at a 400× magnification ratio using a Motic EasyScan Pro digital slide scanner and saved as multilevel image files.

**Figure 2:**
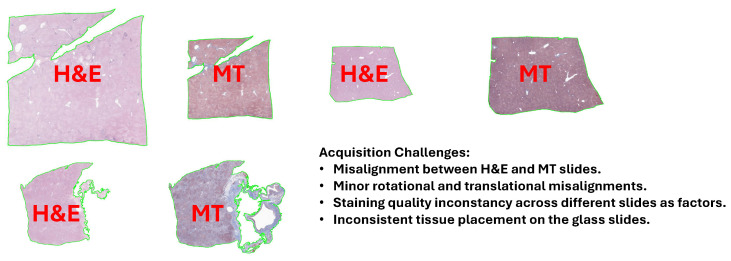
Illustration of key dataset challenges addressed in our study, including misalignment (rotational and translational) between H&E and MT slide pairs, inconsistent staining quality across slides, and variable tissue placement on glass slides.

Challenges: Although the process was meticulously planned, it faced some problems (see Figure 2) during the data collection and pre-processing stages. A key issue was the lack of alignment between the H&E and MT slides as both were prepared from different/consecutive cross-sections of the tissue (they often showed significant spatial discrepancies). Errors made by the humans during the preparation of the slides (for example, differences in the way the tissue was placed on the glass slides and changes in the staining protocols) were also factors that led to misalignment. Moreover, the scanning process induced slight rotational and translational misalignments; therefore, it was difficult to find exact matching between the H&E and MT images. The problems with the alignment became a major issue for the application of fibrosis detection and quantification, which is a process that requires accurate spatial correspondence between the two stains [7,23,24,25].

Another issue was that the staining quality varied from one slide to another, and the reasons behind these variations included (a) reagent degradation, (b) inconsistent staining protocols, and (c) tissue thickness differences. For example, some MT slides had certain regions that looked over-stained or under-stained, while some other MT slides had artifacts like tissue folding or air bubbles. This made it difficult to standardize the input WSI data for the virtual staining model and thus necessitated pre-processing steps (like stain normalization and artifact removal) [26,27,28]. Moreover, the enormous size of WSIs led to computational problems as WSIs usually have dimensions of more than 100,000 × 100,000 pixels, and thus performing operations on them directly is computationally very expensive.

Solution: We addressed those challenges through the development of a detailed pre-processing pipeline (described in detail in the following subsections and summarized in Table 2), which included tile extraction, rigid-body registration, and FFD. It guaranteed (after evaluation) that H&E and MT slides are in correct spatial alignment even when staining variability and misalignments are present.

### 2.2. Tiles Extraction and Alignment

The pre-processing pipeline begins by using the WSIs to identify regions of interest (ROIs) and extract contours that define the tissue boundaries. As noted, WSIs are typically extremely large, and hence thumbnails are generated by downsampling those WSIs. The downsampling factor is carefully chosen to balance computational efficiency with the preservation of structural details. For instance, a scale factor that reduces the image dimensions to 1024×1024 pixels can be sufficient for initial processing. After that, contours are extracted to delineate the tissue regions. This is achieved by (1) converting the thumbnail to grayscale, (2) applying Gaussian blur to reduce noise, and (3) using Otsu’s thresholding to create a binary image. The grayscale conversion is applied using the weighted sum of the RGB channels using I(x,y)=0.299×R(x,y)+0.587×G(x,y)+0.114×B(x,y), where R(x,y), G(x,y), and B(x,y) are the red, green, and blue channels of the image, respectively.

Gaussian blur is then applied to smooth the image using $I_{\mathrm{blur}}\left(x,y\right)=I\left(x,y\right)*G\left(x,y;\sigma \right)$ where G(x,y;σ) is the Gaussian kernel with standard deviation σ, and * denotes convolution. The Gaussian kernel (G(x,y;σ)) is defined as $\left(\frac{1}{2\times \pi \times \sigma^{2}}\right)\times \exp\left(-\frac{x^{2}+y^{2}}{2\times \sigma^{2}}\right)$. Otsu’s thresholding is used to create a binary image B(x,y) using Equation (1) where the threshold is determined by minimizing the intra-class variance using $\arg\min_{t}\left(\omega_{1}\left(t\right)\times \sigma_{1}^{2}\left(t\right)+\omega_{2}\left(t\right)\times \sigma_{2}^{2}\left(t\right)\right)$ [29]. In it, $\omega_{1}\left(t\right)$ and $\omega_{2}\left(t\right)$ are the probabilities of the two classes (foreground and background) separated by threshold *t*, and $\sigma_{1}^{2}\left(t\right)$ and $\sigma_{2}^{2}\left(t\right)$ are their variances.

(1) $$B\left(x,y\right)=\left\{\begin{array}{cc} 1 & \mathrm{if}\;I_{\mathrm{blur}}\left(x,y\right)>\mathrm{Threshold}\\ 0 & \mathrm{Otherwise} \end{array}\right.$$

The largest contour *C* (corresponding to the main tissue region) is selected as the ROI using $C=\arg\max_{\mathrm{contour}}\mathrm{Area}\left(\mathrm{contour}\right)$. This step ensures that non-tissue regions, such as glass slides or artifacts, are excluded from further analysis. With the contours extracted, the next step is to match the H&E and MT thumbnails using the SIFT algorithm [30,31]. This ensures that the slides can be used for further processing if the matching percentage is above a certain threshold (empirically selected). SIFT detects keypoints and computes descriptors that are invariant to scale, rotation, and illumination changes. The keypoints and descriptors for the H&E and MT thumbnails are computed as in Equation (2). The descriptors from the H&E and MT images are then matched using a brute-force matcher. The Euclidean distance between $\left(d_{1},d_{2}\right)$ is used to measure the similarity between descriptors by applying $\sqrt{\sum_{i=1}^{n}{\left(d_{1,i}-d_{2,i}\right)}^{2}}$.

(2) $$\begin{array}{cc} {\mathrm{Keypoints}}_{H\&E},{\mathrm{Descriptors}}_{H\&E} & =\mathrm{SIFT}({\mathrm{Thumbnail}}_{H\&E})\\ {\mathrm{Keypoints}}_{\mathrm{MT}},{\mathrm{Descriptors}}_{\mathrm{MT}} & =\mathrm{SIFT}({\mathrm{Thumbnail}}_{\mathrm{MT}}) \end{array}$$

**Figure 3:**
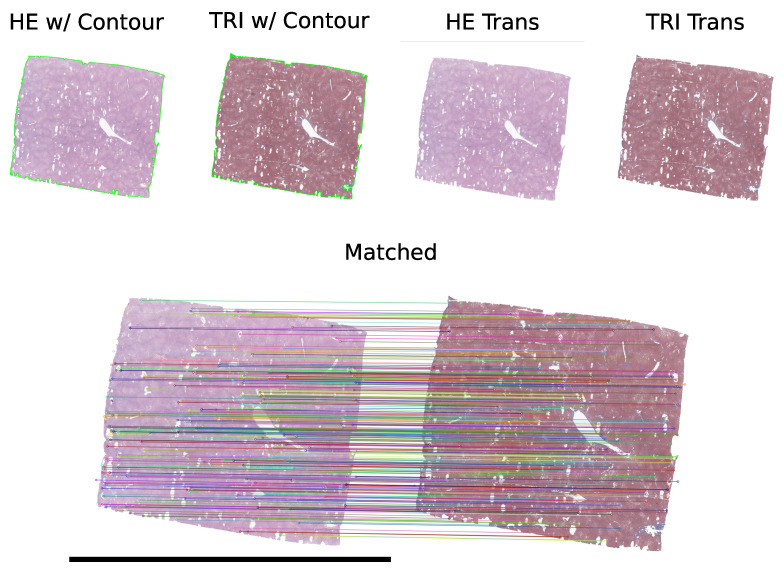
Visualization of the alignment of corresponding tissue regions from H&E- and MT-stained slides using the SIFT algorithm. High-confidence feature matches are highlighted, ensuring accurate spatial correspondence for downstream multi-modal analysis. The lower row refers to the matched H&E and MT thumbnails.

Matches are filtered using a ratio test to retain only high-confidence correspondences. This filtering step is mandatory to ensure that the matches are reliable and not influenced by noise or outliers. A sample of matched H&E and MT thumbnails is shown in Figure 3. Once the H&E and MT thumbnails are matched, the following step is to extract patches from the slides. To do so, the bounding box of the H&E contour is calculated (to define the region of interest), and the center point of the H&E contour is determined. The bounding box is represented by its top-left corner $\left(x_{\min},y_{\min}\right)$ and its width and height (w,h). The center point of the H&E contour is calculated to serve as the reference for patch extraction. The center point is $\left(x_{c},y_{c}\right)$ where $x_{c}$ is $\left(x_{\min}+\frac{w}{2}\right)$ and $y_{c}$ is $\left(y_{\min}+\frac{h}{2}\right)$.

The number of patches per side is determined based on the pre-defined patch size and overlap values. If the patch size is S×S and the overlap is *O*, then the number of patches per side *N* is determined utilizing $N=\left\lfloor \frac{w-O}{S-O}\right\rfloor$ where $\left\lfloor □\right\rfloor$ refers to the floor function. The coordinates $\left(x_{p},y_{p}\right)$ of each patch are calculated using $x_{p}=x_{c}+i\times \left(S-O\right),\;y_{p}=y_{c}+j\times \left(S-O\right)$ where *i* and *j* are indices ranging from $-\frac{N}{2}$ to $\frac{N}{2}$. For each patch location $\left(x_{p},y_{p}\right)$, the corresponding regions are extracted from the H&E and MT slides. This step is iteratively applied through each patch location and extracts the corresponding regions from the H&E and MT slides.

The extracted patches are aligned using the ORB (Oriented FAST and Rotated BRIEF) algorithm and homography transformation. The ORB algorithm detects keypoints and computes descriptors for the H&E and MT patches. The keypoints are detected using the FAST (Features from Accelerated Segment Test) algorithm [32], and the descriptors are computed using the BRIEF (Binary Robust Independent Elementary Features) algorithm [33]. The keypoints and descriptors for the H&E and MT patches are computed using Equation (3). The descriptors from the H&E and MT patches are matched using a brute-force matcher where the Hamming distance is used to measure the similarity between descriptors by applying $\sum_{i=1}^{n}\left(d_{1,i}⊕d_{2,i}\right)$ where $d_{1}$ and $d_{2}$ are binary descriptors, and ⊕ denotes the XOR operation.

(3) $$\begin{array}{cc} {\mathrm{Keypoints}}_{H\&E},{\mathrm{Descriptors}}_{H\&E} & =\mathrm{ORB}({\mathrm{Patch}}_{H\&E})\\ {\mathrm{Keypoints}}_{\mathrm{MT}},{\mathrm{Descriptors}}_{\mathrm{MT}} & =\mathrm{ORB}({\mathrm{Patch}}_{\mathrm{MT}}) \end{array}$$

The matches are filtered using a ratio test to retain only high-confidence correspondences. Once the matches are obtained, a homography matrix *H* is computed using the RANSAC (Random Sample Consensus) algorithm [34,35]. The homography matrix represents a projective transformation that maps points from the H&E patch to the MT patch. The homography matrix is a 3×3 matrix and is presented in Equation (4). The transformation of a point (x,y) from the H&E patch to the MT patch is given by Equation (5) where $\left(x^{\prime },y^{\prime }\right)$ are the transformed coordinates. RANSAC is particularly effective in this context because it robustly estimates the transformation even in the presence of outliers. The RANSAC objective function minimizes the reprojection error using $\mathrm{Error}=\sum_{i}{∥{\mathrm{Keypoints}}_{\mathrm{MT},i}-H\times {\mathrm{Keypoints}}_{H\&E,i}∥}^{2}$.

(4) $$H=\left[\begin{array}{ccc} h_{11} & h_{12} & h_{13}\\ h_{21} & h_{22} & h_{23}\\ h_{31} & h_{32} & h_{33} \end{array}\right]$$

(5) $$\left[\begin{array}{c} x^{\prime }\\ y^{\prime }\\ 1 \end{array}\right]=H\times \left[\begin{array}{c} x\\ y\\ 1 \end{array}\right]$$

The H&E patch is transformed using a homography matrix so that it can be in line with the MT patch. The transformed patch (${\mathrm{Patch}}_{H\&\mathrm{E\_aligned}}$) is obtained by $\mathrm{WarpPerspective}({\mathrm{Patch}}_{H\&E},H)$ where WarpPerspective changes the coordinates of the patch according to the homography. Various checks are also done to make sure the extracted and aligned patches are of good quality. The very first step is to find those patches with too many empty regions and then to not consider them. This is achieved by ascertaining the portion of black or white pixels in the patch and checking it against a certain threshold value. The percentage of empty regions is computed by $\left(\frac{\#\;\mathrm{BlackPixels}+\#\;\mathrm{WhitePixels}}{\#\;\mathrm{TotalPixels}}\right)$. For instance, patches with more than 30% empty regions are usually rejected. Afterward, the resemblance between the H&E and MT patches is measured by means of such parameters as mutual information (MI), cosine similarity, and perceptual hash (pHash).

The MI quantifies the statistical dependence between the two images (MI(X,Y)) using H(X)+H(Y)−H(X,Y) where H(X) and H(Y) are the marginal entropies, and H(X,Y) is the joint entropy. The entropy H(X) is calculated using $-\sum_{i=1}^{n}p\left(x_{i}\right)\times \log\left(p(x_{i})\right)$ where $p(x_{i})$ is the probability of intensity $x_{i}$ in the image. Cosine similarity (CosineSimilarity(A,B)) measures the alignment of their intensity distributions using $\frac{A\times B}{∥A∥∥B∥}$ where *A* and *B* are the flattened image vectors. Perceptual hash provides a measure of structural similarity based on image hashes (pHash(A,B)) using HammingDistance(Hash(A),Hash(B)). Patches that do not meet the pre-defined similarity thresholds (i.e., MI ≥0.35, cosine similarity ≥0.75, pHash ≤20) are discarded to ensure that only high-quality, well-aligned patches are retained for further analysis. The final step involves saving the validated patches for further analysis. The H&E and MT patches are saved separately, along with metadata such as their coordinates and similarity scores.

### 2.3. Free-Form Deformation (FFD)

Free-form deformation (FFD) is an effective method that changes the shape of images (i.e., deform) after fitting the model of transformation to the images. In histopathology, FFD significantly contributes to the alignment of H&E and MT patches very accurately. The reason for this is that our H&E and MT slides are reported to have local deformations most of the time, which is due to differences in tissue preparation. Those slides are usually taken from different cross-sections of the tissue, which brings the problem of alignment. Hence, FFD utilizes a B-spline transformation method that allows smooth and small-area changes; thus, the closest areas in the H&E and MT patches are not only visually but also mathematically aligned [36,37,38].

The role of FFD in histopathology is very significant [39]; unlike global transformations like homography (which are limited to correcting rigid misalignments), FFD has the power to repair those local areas which have been distorted due to tissue shrinkage, folding, or unpaired staining. On top of that, since H&E and MT slides are typically taken from different cross-sections of the tissue, they may not be perfectly aligned, thus resulting in misalignments that cannot be fixed by simple transformation techniques. The FFD method is carried out through a series of steps that are clearly defined, and each step is a contribution to the accurate alignment of H&E and MT patches. The B-spline transformation model is initialized to define the deformation grid, which is the governing factor for the flexibility and localization of the transformation marks the start of the process. Next, image registration is carried out; this is done by aligning the source image to the target image using a similarity metric and an optimization algorithm. The last step is to warp the source image with the help of the computed transformation for it to be in line with the target image.

The B-spline transformation technique is specified by a grid of control points that carry out the deformation of the image [40,41]. The grid size determines the flexibility of the transformation, with smaller grid sizes allowing for more localized deformations. The B-spline transformation T(x,y) is represented as $\sum_{i=0}^{n}\left(\sum_{j=0}^{m}\left(\phi_{i}\left(x\right)\times \phi_{j}\left(y\right)\times c_{i,j}\right)\right)$ where $\phi_{i}\left(x\right)$ and $\phi_{j}\left(y\right)$ are the B-spline basis functions, $c_{i,j}$ are the control points, and *n* and *m* are the dimensions of the grid. The B-spline basis functions are defined recursively using Equation (6) for the zeroth-order basis functions (and using Equation (7) for higher-order basis functions) where *k* is the order of the B-spline. That formulation allows for smooth and continuous deformations, making it ideal for aligning histopathology images.

(6) $$\phi_{i}\left(x\right)=\left\{\begin{array}{cc} 1 & \mathrm{if}\;t_{i}\leq x<t_{i+1}\\ 0 & \mathrm{Otherwise} \end{array}\right.$$

(7) $$\phi_{i}^{k}\left(x\right)=\left(\frac{x-t_{i}}{t_{i+k}-t_{i}}\right)\times \phi_{i}^{k-1}\left(x\right)+\left(\frac{t_{i+k+1}-x}{t_{i+k+1}-t_{i+1}}\right)\times \phi_{i+1}^{k-1}\left(x\right)$$

The registration process aligns the source image to the target image by optimizing the B-spline transformation parameters. The similarity score between two pair images ($\mathrm{MMI}(I_{1},I_{2})$) is measured using the Mattes mutual information (MMI) as it quantifies the statistical dependence between the intensity distributions of the two images; its formula is defined in Equation (8), where p(i,j) is the joint probability distribution of the intensities in the two images, and $p_{1}\left(i\right)$ and $p_{2}\left(j\right)$ are the marginal probability distributions of the intensities in the source and target images, respectively.

(8) $$\mathrm{MMI}\left(I_{1},I_{2}\right)=\sum_{i=1}^{N}\left(\sum_{j=1}^{M}\left(p\left(i,j\right)\times \log\left(\frac{p(i,j)}{p_{1}\left(i\right)\times p_{2}\left(j\right)}\right)\right)\right)$$

The optimization process minimizes the MMI metric utilizing the gradient descent optimizer, which updates the transformation parameters iteratively using $\theta_{k+1}=\theta_{k}-\eta \times \nabla \mathrm{MMI}\left(\theta_{k}\right)$, where $\theta_{k}$ are the transformation parameters at iteration *k*, η is the learning rate, and $\nabla \mathrm{MMI}(\theta_{k})$ is the gradient of the MMI metric with respect to the transformation parameters. That iterative processing ensures that the source image is progressively aligned with the target image. Once the optimal transformation parameters are calculated, the source image is warped to align the target image. That warped image $I_{\mathrm{warped}}$ is determined using $I_{\mathrm{warped}}\left(x,y\right)=I_{\mathrm{source}}\left(T\left(x,y\right)\right)$ where T(x,y) is the B-spline transformation. The warping process ensures that the source image is deformed (correcting local misalignments) to match the target image. This step is mandatory for ensuring that corresponding regions in the H&E and MT patches are accurately aligned, even when the slides are from different cross-sections.

### 2.4. Stain Normalization Using Reinhard’s Method

Stain normalization is a critical pre-processing step in digital pathology to ensure consistency in color appearance across different tissue samples [24,42]. Reinhard’s technique is a widely used method for color normalization that aligns the color distribution of an image domain $D^{\in d_{1}}$ to a target image $D^{\in d_{2}}$ by transforming the color space [43,44]. The first step in Reinhard’s method is to translate the input RGB image to the LAB color space. It separates the lightness (*L*) from the color information (*A* and *B* channels), making it suitable for color normalization duties. The LAB color space is perceptually uniform, and changes in color values correspond to consistent changes in human perception. The LAB channels are then normalized (to ensure they fall within specific ranges) using $L_{n}=\frac{L}{2.55}$ where $L_{n}$ is normalized to the range [0,100], while $A_{n}$ ($A_{n}=A-128.0$) and $B_{n}$ ($B_{n}=B-128.0$) are normalized to the range [−128,127]. After performing the normalization in the LAB color space, the image is converted back to the RGB color space using the inverse transformation (using Equation (9)).

(9) $$L=L_{n}\times 2.55,\;A=A_{n}+128.0,\;B=B_{n}+128.0$$

**Figure 4:**
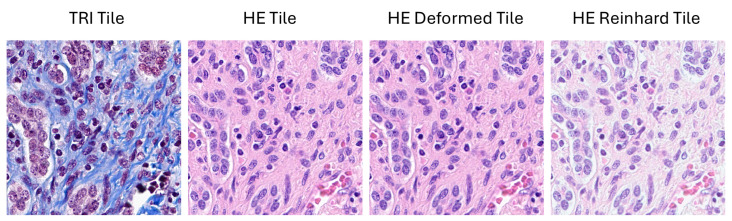
Visualization of stain normalization using Reinhard’s method. It demonstrates the translation of a sample input H&E-stained image into a normalized version using Reinhard’s method. That process aligns the color distribution of the input image to a target image, ensuring consistency in color appearance.

Reinhard’s method involves normalizing an image to match the color distribution of a target image, which is achieved by aligning the mean and standard deviation of the LAB channels of the source image to those of the target image. Mathematically, let $\mu_{L}^{\mathrm{target}}$, $\mu_{A}^{\mathrm{target}}$, and $\mu_{B}^{\mathrm{target}}$ denote the mean values of the *L*, *A*, and *B* channels of the target image, respectively. Similarly, let $\sigma_{L}^{\mathrm{target}}$, $\sigma_{A}^{\mathrm{target}}$, and $\sigma_{B}^{\mathrm{target}}$ denote the standard deviations of the *L*, *A*, and *B* channels of the target image. For the source image, let $\mu_{L}^{\mathrm{source}}$, $\mu_{A}^{\mathrm{source}}$, and $\mu_{B}^{\mathrm{source}}$ denote the mean values of the *L*, *A*, and *B* channels, and let $\sigma_{L}^{\mathrm{source}}$, $\sigma_{A}^{\mathrm{source}}$, and $\sigma_{B}^{\mathrm{source}}$ denote the corresponding standard deviations. The normalized LAB channels of the source image are computed as in Equation (10). The normalized LAB image is then converted back to the RGB color space using the inverse transformation $T_{\mathrm{LAB2RGB}}$. A sample of the stain normalization process using Reinhard’s method is presented in Figure 4.

(10) $$\begin{array}{cc} L_{\mathrm{norm}} & =\frac{\left(L-\mu_{L}^{\mathrm{source}}\right)}{\sigma_{L}^{\mathrm{source}}}\times \sigma_{L}^{\mathrm{target}}+\mu_{L}^{\mathrm{target}}\\ A_{\mathrm{norm}} & =\frac{\left(A-\mu_{A}^{\mathrm{source}}\right)}{\sigma_{A}^{\mathrm{source}}}\times \sigma_{A}^{\mathrm{target}}+\mu_{A}^{\mathrm{target}}\\ B_{\mathrm{norm}} & =\frac{\left(B-\mu_{B}^{\mathrm{source}}\right)}{\sigma_{B}^{\mathrm{source}}}\times \sigma_{B}^{\mathrm{target}}+\mu_{B}^{\mathrm{target}} \end{array}$$

### 2.5. Stain Mapping Using TbGAN

Stain translation is a critical task in computational pathology where the goal is to translate histopathological images from one staining domain to another [24]. The TbGAN (a transformer-based generative adversarial network (GAN)) architecture/network is carefully designed to address that challenge by utilizing the merits of transformers for global feature extraction and GANs for high-quality image generation. It consists of two main components: a generator and a discriminator. The generator is responsible for translating images from one stain domain to another domain, while the discriminator distinguishes between real and generated stain images. Also, the generator incorporates a transformer encoder to capture long-range dependencies in the histopathological image, which is crucial for preserving structural details during stain translation.

The input image $X\in R^{H\times W\times C}$ is split into non-overlapping patches of size (P×P) where *H*, *W*, and *C* represent the height, width, and number of channels of the image respectively. Moreover, each patch is flattened and projected into a high-dimensional embedding space using a learnable projection matrix $W_{e}\in R^{(P^{2}\times C)\times D}$ where *D* is the embedding dimension. After that, the patch embeddings are computed using $Z=X\times W_{e}$ where $Z\in R^{N\times D}$ and $N=\frac{H\times W}{P^{2}}$ is the number of patches. This step transforms the image into a sequence of patch embeddings, which are processed by the transformer encoder.

The transformer encoder processes the patch embeddings using a series of multi-head self-attention (MSA) and feed-forward network (FFN) layers [45,46,47]. The self-attention mechanism computes attention scores between all patches, enabling the model to capture global dependencies in the image. For each patch embedding $z_{i}\in R^{D}$, the self-attention mechanism computes queries Q, keys K, and values V using linear transformations using Equation (11) where $W_{Q},W_{K},W_{V}\in R^{D\times D}$ are learnable weight matrices. The attention scores are computed using (12).

(11) $$Q=Z\times W_{Q},\;K=Z\times W_{K},\;V=Z\times W_{V}$$

(12) $$\mathrm{Attention}\left(Q,K,V\right)=\mathrm{SoftMax}\left(\frac{Q\times K^{T}}{\sqrt{D}}\right)\times V$$

The multi-head mechanism splits the embeddings into *h* heads, computes attention independently for each head, and concatenates the results. The FFN consists of two fully connected layers with a GELU activation function. This is presented in Equation (13) where $W_{1},W_{2}$ are weight matrices and $b_{1},b_{2}$ are biases. Layer normalization is applied before the MSA and FFN layers to stabilize training using $\gamma \times \frac{z-\mu }{\sigma }+\beta$ where μ and σ are the mean and standard deviation of z, and γ,β are learnable parameters.

(13) $$\mathrm{FFN}\left(z\right)=W_{2}\times \mathrm{GELU}\left(W_{1}\times z+b_{1}\right)+b_{2}$$

The output of the transformer encoder is reshaped into a 2D feature map and upsampled using transposed convolutions to reconstruct the image using $Y=\mathrm{ConvTranspose}(Z_{\mathrm{out}})$ where $Z_{\mathrm{out}}$ is the output of the transformer encoder, and Y is the reconstructed image. The discriminator *D* is a CNN that classifies images as real or fake. It consists of convolutional layers with LeakyReLU activations and instance normalization. The output of the discriminator is a probability score and described in D(X)=σ(Conv(X)) where σ is the sigmoid function.

The model is trained using a combined loss function between adversarial, cycle-consistency, and identity losses. The adversarial training framework ensures that the generated images are realistic, while the cycle-consistency and identity losses provide additional constraints to improve translation quality. The adversarial loss encourages the generator *G* to produce realistic images that fool the discriminator *D*. For domain *A* and domain *B*, the adversarial losses are presented in Equation (14) where $p_{A}$ and $p_{B}$ are the data distributions for domains *A* and *B*, respectively.

(14) $$\begin{array}{cc} L_{\mathrm{adv}}^{A} & =E_{X∼p_{A}}\left[\log D_{A}\left(X\right)\right]+E_{Y∼p_{B}}\left[\log\left(1-D_{A}\left(G_{A}\left(Y\right)\right)\right)\right]\\ L_{\mathrm{adv}}^{B} & =E_{Y∼p_{B}}\left[\log D_{B}\left(Y\right)\right]+E_{X∼p_{A}}\left[\log\left(1-D_{B}\left(G_{B}\left(X\right)\right)\right)\right] \end{array}$$

The cycle-consistency loss ensures that translating an image from domain *A* to domain *B* and back to domain *A* results in the original image. This loss is presented in Equation (15). The identity loss ensures that the generator preserves the identity of the input image when no translation is needed. This loss is presented in Equation (16). The total loss is a weighted sum of the adversarial, cycle-consistency, and identity losses. It is described in Equation (17) where $\lambda_{\mathrm{cycle}}$ and $\lambda_{\mathrm{identity}}$ are hyperparameters.

(15) $$\begin{array}{cc} L_{\mathrm{cycle}}^{A} & =E_{X∼p_{A}}[∥X-G_{A}\left(G_{B}\left(X\right)\right){∥}_{1}]\\ L_{\mathrm{cycle}}^{B} & =E_{Y∼p_{B}}[∥Y-G_{B}\left(G_{A}\left(Y\right)\right){∥}_{1}] \end{array}$$

(16) $$\begin{array}{cc} L_{\mathrm{identity}}^{A} & =E_{X∼p_{A}}[∥X-G_{A}\left(X\right){∥}_{1}]\\ L_{\mathrm{identity}}^{B} & =E_{Y∼p_{B}}[∥Y-G_{B}\left(Y\right){∥}_{1}] \end{array}$$

(17) $$L_{\mathrm{total}}=L_{\mathrm{adv}}^{A}+L_{\mathrm{adv}}^{B}+\lambda_{\mathrm{cycle}}\times \left(L_{\mathrm{cycle}}^{A}+L_{\mathrm{cycle}}^{B}\right)+\lambda_{\mathrm{identity}}\times \left(L_{\mathrm{identity}}^{A}+L_{\mathrm{identity}}^{B}\right)$$

In the current study, two emphases were employed in each experiment: one to on $\lambda_{\mathrm{cycle}}$ and the other on $\lambda_{\mathrm{identity}}$. They were designed by scaling the respective hyperparameters by factors ranging from 1 to 3. Emphasizing $\lambda_{\mathrm{cycle}}$ ensures stronger enforcement of cycle-consistency, which is critical for maintaining structural and semantic consistency between the source and target domains during translation. On the other hand, emphasizing $\lambda_{\mathrm{identity}}$ encourages the model to preserve the identity of the input images when no translation is needed, which helps in retaining the original features and reducing unnecessary transformations.

### 2.6. Fusion Mechanism

The weighted fusion mechanism marks a conceptual progression beyond the usual ensemble methods by intentionally utilizing the compensation of orthogonal bias. While typical ensembles combine the outputs models with similar architectures that differ only by random initialization, our fusion takes four configurations (shown in Table 3) with optimization objectives that are fundamentally different, and the failure modes are complementary as follows:

(i) Identity-focused configurations ($\lambda_{\mathrm{identity}}$ emphasis) preserve cellular morphology and structural integrity when minimal translation is required but systematically under-translate subtle collagen structures in fibrotic regions.
(ii) Cycle-consistency-focused configurations ($\lambda_{\mathrm{cycle}}$ emphasis) enforce semantic and structural alignment between source and target domains but tend to over-smooth diagnostic features through excessive transformation.
(iii) Original-input configurations maintain native stain characteristics but exhibit sensitivity to inter-slide staining variability.
(iv) Reinhard-normalized configurations improve stain consistency across batches but may introduce color-space artifacts in heterogeneous tissue regions.

**Figure 5:**
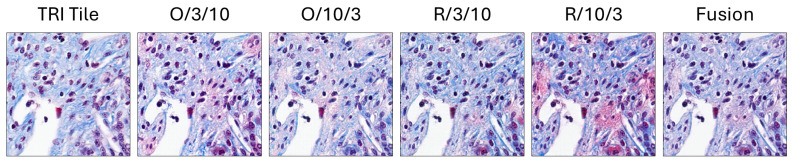
Visualization of the weighted fusion process. It illustrates the fusion of outputs from the four configurations: original with emphasis on $\lambda_{\mathrm{identity}}$, original (short as O) with emphasis on $\lambda_{\mathrm{cycle}}$, Reinhard-normalized (short as R) with emphasis on $\lambda_{\mathrm{identity}}$, and Reinhard-normalized with emphasis on $\lambda_{\mathrm{cycle}}$. The fusion process combines these outputs using a weighted averaging approach, balancing identity preservation and cycle-consistency.

This fusion method intentionally offsets the weaknesses of individual models (a cycle-consistency focused model has an over-transformation tendency, while an identity-preservation focused one has an under-transformation tendency) by harnessing their complementary strengths for improved overall framework performance. The weighted fusion combines outputs from the four configurations using a fixed arithmetic mean using $Y_{\mathrm{fused}}=\frac{1}{4}\times \sum_{k=1}^{4}Y_{k}$ where $Y_{k}$ represents the output of the *k*-th configuration. Weights were fixed at 0.25 following an empirical grid search of 100 combinations, which revealed a broad performance plateau (MI variation < 0.003) around the equal-weight solution. This indicates robustness to minor weight variations, suggesting precise tuning is not critical for performance stability. Most importantly, the fused result is significantly better than all individual configurations (Wilcoxon signed-rank test, p<0.001 for MI, SSIM, NCC) with large effect sizes (Cohen’s d>0.85); thus fusion provides results that are not only the average. The idea of orthogonal bias compensation allows stain translation in histopathology to be done in a robust and generalizable manner while biologically relevant critical diagnostic features are preserved. The full fusion pipeline is shown in Figure 5.

### 2.7. Performance Evaluation

To evaluate the performance of the proposed H&E-to-MT conversion framework, multiple metrics were employed at the patch level. These metrics quantify the similarity between the generated MT images and the ground truth MT images, with results aggregated via mean and standard deviation across trials. They are categorized into similarity metrics (where higher values reflect better performance) and dissimilarity metrics (where lower values reflect better performance) [48,49]. They are useful for comparing the two images (i.e., original MT and generated MT) as they provide observations into structural alignment, color consistency, texture preservation, and perceptual quality [50,51,52]. Table 4 describes those metrics along with their mathematical formulations, categories, benefits, and insights.

## 3. Experiments and Discussion

### 3.1. Assessment Configurations

As summarized in Table 5, the experiments were managed on a Windows 11 device with a 6 GB GPU (NVIDIA RTX A2000) and 256 GB of RAM. The implementation was developed using Python v3 with the PyTorch v.2.7.1 package serving as the principal DL framework. The dataset consisted of 27 WSIs split into 80% patients for training and 20% patients for testing. Each WSI underwent the pre-processing pipeline that is described in Section 2, including tile extraction, alignment, FFD, and filtration to ensure high-quality input data for the stain translation process. For the stain translation training process, the Adam optimizer was occupied for weight optimization with a learning rate of $10^{-5}$ and beta values set to $\beta_{1}=0.5$ and $\beta_{2}=0.999$. Those hyperparameters were chosen to balance between the convergence speed and stability during training. The input patches were resized to 256 × 256 × 4 (RGBA) dimensions (following empirical trade-off analysis between contextual field-of-view and transformer memory constraints). Pixel values were normalized to [−1,1] to improve training stability. As discussed earlier, four distinct experiment configurations (refer to Table 3) were designed to evaluate the performance of the proposed framework and to achieve a weighted fusion between their pair outputs.

### 3.2. Liver Specimen Assessment (Quantitative and Qualitative)

The quantitative and qualitative studies delineate the assessment of the suggested model; they essentially entail the comparison of the four single configurations’ (i.e., O/3/10, O/10/3, R/3/10, and R/10/3) performances with that of the merged/fused configuration. “O” refers to the original tile while “R” refers to the Reinhard-normalized version. The first value (i.e., 10 or 3) reflects the $\lambda_{cycle}$, while the second reflect the $\lambda_{identity}$. The results conveyed show the fusion process’s success in being able to locate the most accurate and hence reliable stain translation.

#### 3.2.1. Quantitative Analysis

The quantitative statistics (refer to Table 6) provide a comprehensive comparison between the four configurations and the fused method over 24 various trials; the statistics that are reported refer to similarity (↑) and dissimilarity (↓) metrics along with their mean and standard deviation values that give an insight into the consistency and reliability of the results. The fusion strategy showed the maximum average values for the similarity metrics such as MI (0.9815), SSIM (0.7474), and NCC (0.9320), which indicate that the most accurate spatial and color matching were achieved in the fusion mechanism as compared to the individual configurations. Looking into the dissimilarity metrics, the fusion reported the lowest average values for MSE (63.8018), NMSE (0.1359), and GMSD (0.2009); thus, it is clear that the pixel-wise errors are near to zero, and the perceptual quality is high. Moreover, the fusion model is close to the standard deviations for most of the metrics (e.g., SSIM (Std=0.0597) and NCC (Std=0.0220)), thus indicating that it has an acceptable degree of stability and trustworthiness. In particular, the “O/3/10” setup is very good at identity retention (e.g., MI: 0.9460), whereas the “R/10/3” setup is somewhat weaker in most similarity metrics and still can ensure the stability of the cycle (e.g., SSIM: 0.7102). The fusion mechanism is an optimal way to use the advantages of four different settings, thus resulting in a powerful and generalizable method that is insensitive to variations in tissue structure and staining quality, as can be seen from the performance measures such as HistInt (0.8733) and UQI (0.9286).

**Figure 6:**
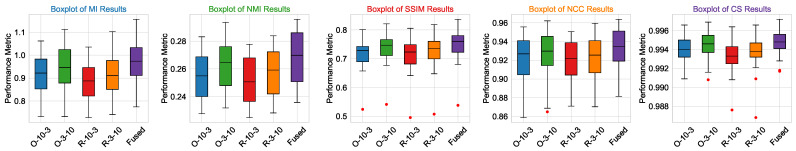
Box plots illustrating the distribution of key similarity metrics (MI, NMI, SSIM, NCC, and CS) across the four individual configurations (O/3/10, O/10/3, R/3/10, and R/10/3) and the fused configuration over 24 trials. Each box represents the IQR of the metric values, with the median indicated by the central line. Whiskers extend to the minimum and maximum values within 1.5 times the IQR, and outliers are shown as individual points. The fusion configuration demonstrates consistently tighter IQRs and fewer outliers across all five metrics, indicating superior consistency and reliability in preserving structural alignment, color fidelity, and statistical similarity.

The box plots depicted in Figure 6 visually summarize the 24 distinct trial performance distributions for each configuration and concentrate on the similarity metrics of the highest importance: MI, NMI, SSIM, NCC, and CS. The fusion setup demonstrates less spread of the interquartile ranges (IQRs) for the majority of these metrics, thus implying that the variability is decreased and that the reliability is increased. As an illustration, the fusion IQR for MI is narrower than those of all the other single configurations, thus illuminating the stability of the mutual information preservation. Along the same lines, the fusion exhibits more compressed distributions for NCC and CS, thus indicating fewer extreme values and more consistent performance across trials.

**Figure 7:**
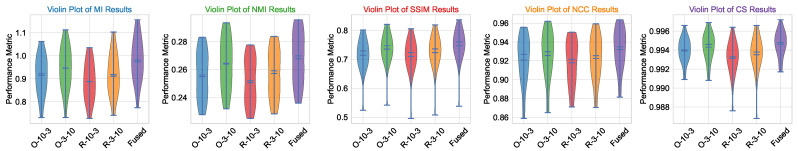
Violin plot illustrating the distribution of key similarity metrics (MI, NMI, SSIM, NCC, and CS) across the four individual configurations (O/3/10, O/10/3, R/3/10, and R/10/3) and the fused configuration over 24 trials where each violin shape represents the kernel density of metric values, with width corresponding to the relative frequency of observations. The fusion configuration exhibits taller and narrower violins across all five metrics, indicating a higher concentration of results near the median.

The violin plot (see Figure 7) is used to give additional support to the box plots, and indeed it shows the data distributions behind the different patterns in the data in a more visual and immediate way. The fusion method was able to produce more accurate results as is evidenced by the fact that it has narrower and higher violins for MI, NMI, SSIM, NCC, and CS; thus it is suggested that the more the data points are concentrated around the median, the more consistent the data is. In fact, this representation is very useful if one wants to find small details of the distribution such as skewness or bimodality, which can be hidden in the box plots. Thus, accompanied by the second visual, the overall message of the paper is that the fusion method can give dependable and steady results in all similarity metrics and for all experimental trials.

**Figure 8:**
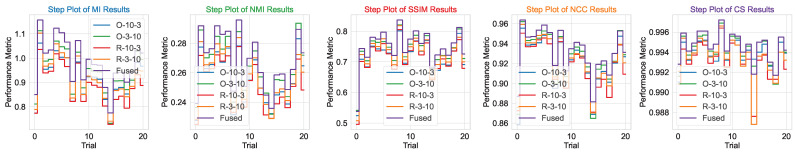
Step plots showing the trial-wise performance across 24 experiments for five key similarity metrics (MI, NMI, SSIM, NCC, and CS) where each subplot tracks the metric values for the four individual configurations (O/10/3, O/3/10, R/10/3, and R/3/10) and the fused configuration over successive trials. The fused approach consistently reports higher metric values with smoother trajectories, indicating stable and superior performance across all trials and metrics.

Moreover, Figure 8 shows step plots that depict the trial-wise changes in each metric throughout the 24 experiments. Each subplot represents one of the five metrics (MI, NMI, SSIM, NCC, and CS) and illustrates that the fused configuration is consistently achieving higher performance with smoother, less erratic trajectories compared to the individual configurations. This temporal stability thus highlights the fusion’s trustworthiness over the repeated trials.

**Figure 9:**
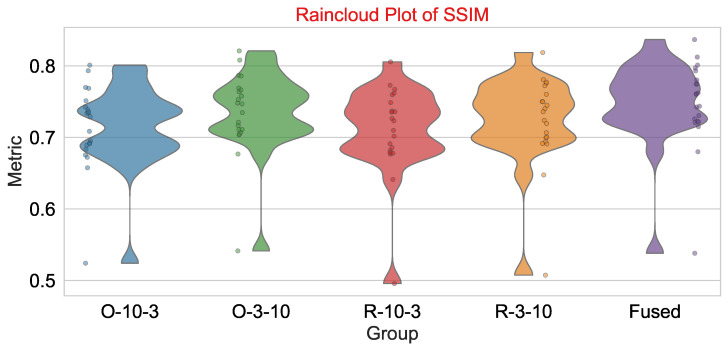
Raincloud plot of SSIM performance across the four individual configurations (O/10/3, O/3/10, R/10/3, and R/3/10) and the fused configuration over 24 trials. The plot combines a box plot (interquartile range and median), raw data points (rain), and a smoothed density distribution (cloud). The fused configuration shows a higher median SSIM (0.7474), tighter spread, and greater density around the peak, confirming its enhanced consistency and reliability in preserving structural similarity.

Lastly, Figure 9 offers an elaborate raincloud plot just for SSIM, combining raw data points (“rain”), a box plot (IQR and median), and a smoothed density (“cloud”). The fused configuration attains the highest median SSIM (0.7474) and displays the closest grouping of values around this median, with very few outliers; thus, it is a demonstration of structural similarity preservation at a high level and of great consistency.

As a result, those figures together are a constant confirmation that the fusion method is not only superior in average metric values to individual configurations but also has higher robustness, lesser variance, and more stable behavior in all the 24 trials for the core similarity metrics of interest.

#### 3.2.2. Statistical Validation Framework

To evaluate the performance differences among the five approaches (four individual configurations plus fused output) while utilizing n=1 (batch size) per inference, we conducted a one-way repeated-measures ANOVA on each similarity metric across 24 independent trials. Significant main effects were observed for all metrics, and they confirm the meaningful performance variation across approaches: MI: F(4,92)=190.89, p<0.001; NMI: F(4,92)=124.41, p<0.001; SSIM: F(4,92)=138.46, p<0.001; NCC: F(4,92)=45.92, p<0.001; CS: F(4,92)=17.07, p<0.001 where 92 resulted from (k−1)×(n−1)=4×23. Tukey’s Honest Significant Difference (HSD) post hoc tests with Bonferroni correction revealed that the fused approach significantly outperformed all individual configurations (all pairwise comparisons p<0.001). Table 7 presents 95% confidence intervals for mean differences between fused and individual configurations.

The Anderson–Darling test confirmed that all metrics had different distributions across configurations (p<0.05); however, the Shapiro–Wilk test results were mixed (normal for MI/NMI/NCC and non-normal for SSIM/CS-R-3/10). Since the Anderson–Darling test is more sensitive to tail deviations in small samples (n=24), we decided to use non-parametric testing. Wilcoxon signed-rank tests revealed that the fused output was significantly better than each individual configuration (all p<0.01, with 19/20 comparisons p<0.001) and that the effect sizes were large (Cohen’s d≥0.77 for all metrics; see Table 8). Most importantly, the fused method exhibited stable signal improvement varying from 0.02% (CS vs. best config) to 9.73% (MI vs. worst config), thus delivering a solution to the n=1 problem through algorithmic robustness. The uniformity of improvements in all 24 trials (100% win rate) is another piece of evidence that the result is valid and not simply due to variance in the experiment.

#### 3.2.3. Qualitative Assessment

**Figure 10:**
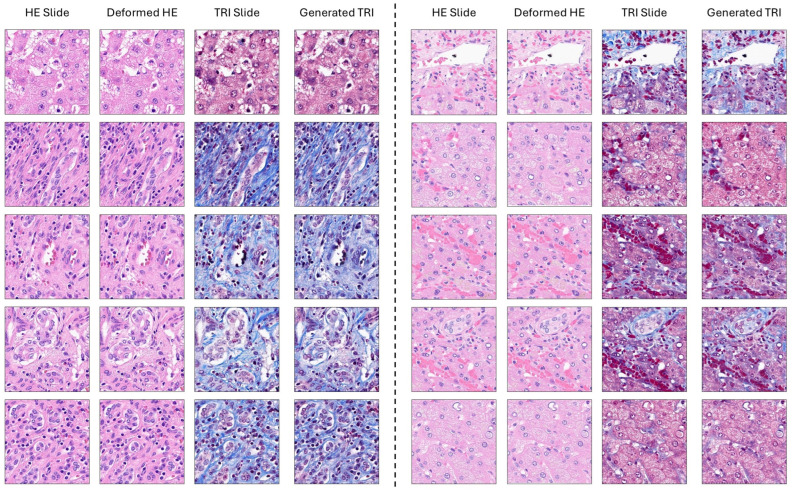
Qualitative analysis of the proposed framework: sample outputs for 10 cases. For each case, there are four images: H&E slide, deformed H&E slide (aligned using FFD), MT slide (ground truth), and generated MT slide. The findings illustrate the system’s capability to correctly convert H&E-stained images to virtual MT-stained images while maintaining not only the structural alignment but also the color and texture details. The synthesized MT images look very much like the real ones, which is an indication that the proposed method is quite successful.

Qualitative outcomes (refer to Figure 10) exemplify the framework’s efficiency through the visuals. For the 10 different cases, the framework displays (a) H&E slide, (b) deformed H&E slide (aligned using FFD), (c) MT slide (ground truth), and (d) generated MT slide. First, we notice that the synthetic MT slides look very much like the real MT slides; the correct representation of collagen fibers (blue areas) and cellular structures (red areas) can be seen visually. Ensuring precise alignment between the MT slides and the H&E slides by reflecting on the deformations applied to the H&E slides, it additionally supports the quality of the created images. Mainly, it is in the case of complex tissue architectures or uneven/irregular staining that this is manifested as the framework handles the changes in tissue structure and staining quality perfectly.

As an example, in the case of uneven staining or artifacts, the generated MT slides are in good agreement with the ground truth; thus, the framework’s stability is demonstrated. The fusion setting, on the other hand, keeps the minutest parts such as tiny collagen fibers and cellular boundaries that are necessary for correct fibrosis assessment. This is a huge step forward from the individual configurations (which may over-smooth or distort these details). The qualitative inspections reveal the potential of the framework to create realistic virtual MT images from H&E-stained slides, thus, opening new possibilities in fibrosis assessment and beyond in histopathology.

#### 3.2.4. Overall Assessment Insights

The chosen set of quantitative measures (combined with qualitative evaluation) offers a thorough appraisal of the proposed model. Notably, it was observed that the fusion method surpassed single setups in both similarity and dissimilarity metrics (refer to Table 6); hence, it was able to reconcile the identity preservation with cycle-consistency. That makes the proposed framework a reliable and a broadly applicable approach for stain translation in histopathology. The fusion mechanism shows less variability from different trials as can be seen from its more compact IQRs and lower standard deviations. Such consistency is absolutely necessary for clinical applications where it must be noted that reliability is of utmost importance.

The ability of the framework to produce high-quality virtual MT images from H&E-stained slides opens up many possibilities for fibrosis assessment and numerous other histopathological applications. It is a method that does away with the necessity of physical re-staining; thus, it saves time and money, and at the same time, it improves diagnostic workflows. The effectiveness of the fusion mechanism raises the point that further fine-tuning of weighting schemes or the addition of supplementary configurations might lead to better results. In addition, the potential of the framework could be realized by simply extending it to other stains translation (e.g., H&E to immunohistochemistry), which in turn could increase its applicability.

### 3.3. Medical Relevance and Clinical Validity

The framework we have presented is highly relevant in a clinical context, particularly for digital pathology workflows that, among other things, depend on precise cross-stain alignment for diagnostic accuracy, computational biomarker quantification, and patient monitoring over time. Our detailed analytical results reflect the top technical capability of the system (which includes less variability that is shown by the results from 24 trials and similarity metrics (MI, SSIM, NCC, and CS) that are consistently higher); however, clinical validity is more than just a matter of metrics, and it requires an understanding of the impact of diagnostics in the real world.

Our tool is still able to produce diagnostically relevant images even when faced with typical issues in histopathology such as staining variations, slight tissue folding, and sectioning defects. A qualitative evaluation (Figure 10) shows that the morphologic features of collagen necessary for fibrosis grading have been well retained visually. Nevertheless, we have to recognize the inability to deal with very problematic cases such as surface areas with heavy hemorrhage, large folding, or tissue breaks; the assumption here is that pre-processing quality control is a must before virtual staining, not a limitation to physical restaining protocols only. Most important is the fact that, being unsupervised, the model does not require adaptation or recalibration at every new institution or for each patient; therefore, this method solves one of the major problems for clinical implementation that supervised methods face, i.e., dependence on site-specific training data.

From a clinical integration perspective, the framework enables three high-value applications:

- Multi-stain co-registration: Precise alignment of H&E with immunohistochemistry (IHC) or fluorescence stains facilitates spatial biomarker analysis and co-localization studies without physical resection.
- Tissue microarray harmonization: Batch-effect mitigation across hundreds of cores enables reliable quantitative comparisons in high-throughput biomarker studies.
- Longitudinal monitoring: Consistent virtual staining of serial biopsies from the same patient supports objective tracking of fibrosis progression/regression during therapeutic intervention.

Various statistical tests also reinforce the level of clinical readiness: (i) smaller deviations and IQRs over several metrics indicate that the method is resistant to technical noise and biological variations; (ii) no outliers in the fused configurations allow the method’s unsupervised deployment in high-throughput situations; and (iii) the method’s very strong pixel-level matching (CS ≈ 0.9946) guarantees that it can be trusted for those downstream operations that require exact spatial matching (e.g., cell segmentation, nuclei counting, and tumor margin delineation). The framework is designed to be strictly a tool that supports the decision of a pathologist and enhances (not substitutes) the pathologist’s expertise. Its modular design allows the platform to be integrated easily into the existing digital pathology ecosystems (e.g., QuPath) without changing the current diagnostic workflows, thus making it a viable option for those diagnostics to be performed at home without staining the tissues and also for situations where tissue preservation is a priority.

### 3.4. Ethical Considerations and Implementation

Using automated image alignment techniques in medical diagnostics models based on machine/deep learning raises various ethical, technical, and legal issues. In particular, this section outlines the ethical charter under which this research was conducted, discloses the stages of clinical integration, and explains how algorithmic decision-making is reproducible and transparent.

Ethical Guidelines and Patient Data Privacy: The experiments were carried out on anonymized histopathological images, whose use was approved by the IRB (IRB: 22.0448). During model development, including training, testing, and visualizing, no patient information that could identify individuals was saved, used, or communicated. The fusion technique is based exclusively on pixel-level intensity and morphological characteristics and therefore does not attempt to extract or store sensitive phenotypic, genomic, or demographic information, thereby lowering the risk of algorithmic bias or accidental disclosure of data. All pre-processing steps (e.g., stain normalization and spatial registration) were conducted in secure, access-controlled premises compliant with HIPAA, GDPR, and other regional health data regulations. Data retention policies follow the “data minimization” principle: raw images are removed after feature extraction, and only consolidated performance metrics (e.g., mean, std, and IQR) are retained for reporting.

Clinical Deployment and Workflow Integration: In order to promote usage outside the laboratory, the newly developed fusion method has been conceived as a modular, minimally invasive, and detachable post-processing unit able to work with many standard digital pathology platform (for example, QuPath) after configuration. Its integration is possible with:

- Multi-stain co-registration pipelines: Aligning H&E with immunohistochemistry (IHC) or fluorescence stains for spatial biomarker analysis.
- TMA workflows: Harmonizing core-to-core alignment across hundreds of tissue spots to ensure consistent quantification.
- Longitudinal patient monitoring: Tracking morphological changes over time by aligning serial biopsies from the same patient.

The system requires no retraining for new tissue types or staining protocols due to its unsupervised, similarity-driven design, making it highly adaptable to diverse clinical settings without compromising performance consistency.

Regulatory Pathway and Clinical Validation: This approach is conceived as a supportive tool to pathologists; hence, it is not a direct substitution for human diagnostic capabilities. Consequently, it is regulated as a Class II medical device in most locations of the world (e.g., FDA 510(k), CE Marking according to MDR). It is our suggestion to confirm the effectiveness through prospective multi-center validation studies with expert reviewers blinded to the study design. Embedding ethical safeguards, ensuring algorithmic transparency, and giving clinical usability the first priority, this fusion method is a step away toward trustworthy, scalable, and fair AI-assisted pathology workflows.

### 3.5. Empirical Profiling and Computational Costs

Model complexity was empirically profiled using PyTorch memory instrumentation tools to ensure clinical deployability under realistic hardware constraints. As shown in Table 9, the TbGAN architecture comprises 31.3 million parameters (28.5 M transformer generator and 2.8 M CNN discriminator) with generator inputs processed through 8 × 8 patch embedding kernels yielding a sequence length of $N={\left(256/8\right)}^{2}=1024$ tokens per image. This configuration results in quadratic attention complexity ($O(N^{2})$) with $N^{2}=1,\;048,\;576$ operations per layer, consuming 384 MB for attention matrices alone during training.

Combined with parameter storage (108.78 MB), gradients (108.78 MB), optimizer state (217.57 MB), and activations (289 MB), peak training memory reached 1253 MB (20.9% of our 6 GB GPU capacity). This enabled stable batch size = 1 operation with gradient accumulation over eight virtual batches to simulate effective batch size = 8 while maintaining diagnostic-grade output fidelity. Training required 68.3 h for 50 epochs on an NVIDIA RTX A2000 (6 GB VRAM). Inference latency averages 1.8 s per tile; total WSI processing time varies by tissue area (approximately 15:20 min per WSI for typical liver biopsies).

The 256 × 256 input size represents the optimal balance between contextual field-of-view and transformer memory constraints. Profiling at multiple resolutions provides irrefutable evidence of $O(N^{2})$ scaling that necessitated this design choice:

- 256 × 256 inputs: *N* = 1024 tokens → 384 MB attention memory (feasible).
- 512 × 512 inputs: *N* = 4096 tokens → 6144 MB attention memory (exceeds clinical GPU constraints).
- 1024 × 1024 inputs: *N* = 16,384 tokens → 98,304 MB attention memory (infeasible without specialized hardware).

This quadratic complexity constraint explains our batch size = 1 configuration and validates the architectural trade-off between contextual fidelity and computational feasibility for fibrosis assessment in resource-constrained clinical environments.

### 3.6. Clinical Workflow Integration

**Figure 11:**
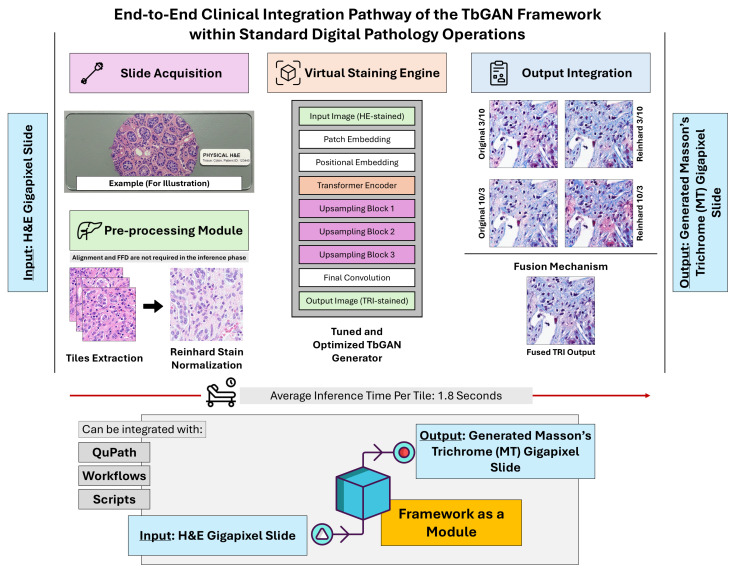
End-to-end clinical integration pathway of the TbGAN framework. The modular architecture transforms H&E slides into diagnostic-quality virtual MT images in 1.8 s per tile without disrupting existing digital pathology workflows or requiring additional tissue sections.

The TbGAN framework has been proposed to work as a modular component showing strong potential for integration into existing digital pathology workflows without disturbing the diagnostic protocols already in place. As depicted in Figure 11, the system is designed as a non-invasive post-processing module that can be used at different stages of a standard digital pathology pipeline. The integration of TbGAN can be initiated at the slide acquisition point where H&E-stained slides are conventionally scanned using normal digital pathology scanners to generate gigapixel WSIs. These WSIs are first processed by our alignment and normalization module that takes care of the important step of spatial registration between H&E and reference MT slides (although this alignment and FFD steps are not necessary at the inference time). Afterward, the virtual staining engine receives the pre-processed tiles to change the H&E style into an MT one by a transformer-based generator through patch embedding, positional encoding, transformer encoding, and upsampling steps in a sequence.

The output integration stage of the framework is characterized by a highly complex fusion mechanism which effectively combines the outputs of multiple configuration variants into a single, high-resolution virtual MT image. This final output can be optimized to be compatible with standard digital pathology platforms like QuPath, which can apply their built-in fibrosis scoring algorithms directly on the virtual MT images without any need for adjustments. Thus, the chain from physical slide to diagnostic result is completed practically in 1.8 s per tile, with hardly any manual input needed.

Perhaps most importantly, the architecture of the system as an independent module allows its use without the necessity of retraining or recalibration when dealing with different tissue types or staining protocols. Pathologists thus have the freedom to operate their familiar digital pathology platforms and at the same time have the benefit of virtual MT images that accurately reflect the fibrosis level. This method solves the main drawback of physical MT staining (i.e., the need for extra tissue sections) while keeping the diagnostic-grade structural fidelity. Moreover, the work process’s speed and the factuality of the use of existing platforms make it a sensible option for resource-limited settings where physical MT staining is not available or tissue conservation is the main consideration; hence, the precision of fibrosis assessment in chronic liver disease is ultimately improved.

**Figure 12:**
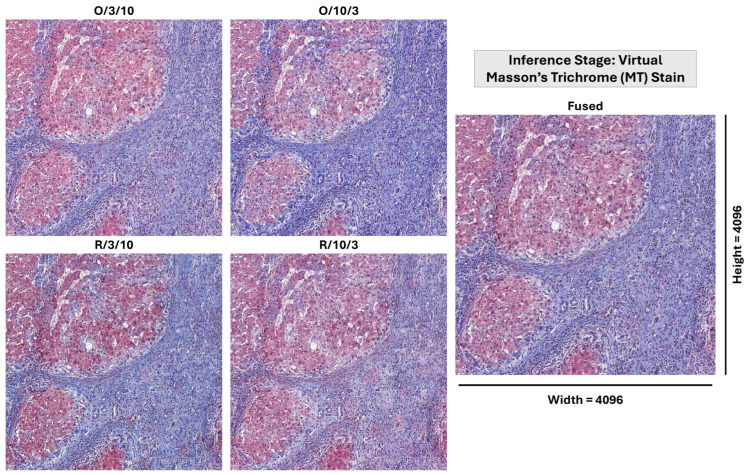
Inference-stage output of the TbGAN framework showing virtual Masson’s Trichrome (MT) generation from H&E input across diverse fibrosis stages. The system preserves collagen morphology (blue) and cellular structures (red) without requiring alignment or FFD during inference.

That said, in clinical deployment, the TbGAN operates entirely during inference without dependence on the multi-stage alignment pipeline, demonstrating its readiness as a standalone virtual staining module (see Figure 12).

## 4. Limitations and Future Directions

A major limitation of the study is a relatively small sample of 27 patients with IRB consent (54 WSIs), which is an outcome of the real-world difficulties of acquiring paired H&E/MT specimens under very strict ethical conditions. It is a fact that tissue in vivo is limited especially for liver biopsies where the tissue has to be preserved, and in this case, obtaining consent from patients for research involves considerable time overhead for specimen collection. In the meantime, we deployed three methodological measures to alleviate concerns over the generalizability of the results:

First of all, our patch extraction process emphasized diagnostic quality at the expense of quantity. Initially, we could have extracted about 1 million patches from the raw WSIs, but after the rigorous exclusion of non-informative regions (>30% background pixels), artifacts (tissue folds, air bubbles), and misaligned pairs (MI < 0.35, cosine similarity < 0.75), only >100,000 high-fidelity patches that effectively reflected morphological changes along the METAVIR fibrosis stages (F0:F4) and tissue architectures were preserved. By prioritizing quality, we ensured that the training signals were derived from valid and relevant diagnostic content rather than from noise.

Secondly, we made sure there was a sharp distinction of patient-level partitioning between training and testing, which means that we did an 80%/20% split at the patient and not image level. By doing so, we stopped our model from identifying patient-specific morphological features or scanner artifacts. This method ensures that the evaluation is a genuine reflection of the model’s ability to generalize to new patients and not a case of overfitting to the images. Thirdly, a statistical test of 24 independent experiments showed that the performance distributions were very good fits to parametric models (Weibull_min, p>0.94) and had narrow 95% confidence intervals (e.g., fused MI: [0.9668,0.9967]), thus revealing consistent behaviors beyond the training cohort.

To address this limitation prospectively, we have initiated collaborations with multiple academic medical centers (including the University of Florida) to expand our validation cohort. Multi-center prospective studies with diverse patient populations and staining protocols are planned as the next translational step toward clinical deployment.

## 5. Conclusions

The proposed virtual H&E-to-MT staining framework is a major breakthrough that significantly contributes to digital pathology especially for fibrosis assessment in chronic liver diseases. By means of TbGAN and the pre-processing pipeline that was discussed, we have managed to overcome the limitation of traditional staining methods, which are misalignment, staining variability, and computational challenges. The fusion method of various settings (a compromise between identity preservation and cycle-consistency) guarantees a robust and precise stain change; this is shown by both the quantitative metrics and the qualitative analysis that have been done. The technology is capable of producing excellent virtual MT images out of H&E-stained slides, and thus physical re-staining will be minimal; the time, the resources and thus the diagnostic workflows will be efficiently utilized. The quantitative results that have been reported showed that the fused configuration is the one that achieved the highest mean values for the similarity metrics (MI: 0.9987, SSIM: 0.7618) and the lowest mean values for the dissimilarity metrics (MSE: 63.3860, GMSD: 0.1987); this indicates that there is a better structural alignment and color consistency. The qualitative analysis that was reported also served as further proof of the framework’s performance where the synthesized MT images displayed prominent visual similarity to the ground truth (i.e., accurate depiction of the collagen fibers and cellular structures). Therefore, the proposed system not only integrates fibrosis assessment functionalities but can still be stretched out to other histopathological areas such as tumor detection and biomarker localization by offering a dependable and accurate method for virtual stain translation. However, as this study was validated on a single-institution cohort, future multi-center trials are required to confirm generalizability across diverse staining protocols and scanner types.

## Figures and Tables

**Table 1:** Comparative analysis between Naglah et al.’s cGAN approach [22] and our TbGAN framework across architectural design, alignment handling, and clinical applicability.

Feature	Naglah et al. (cGAN) [22]	Proposed TbGAN
Architecture	U-Net with skip connections (CNN-based)	Transformer encoder + CNN decoder (hybrid)
Context modeling	Local receptive fields (max 7 × 7 kernel)	Global self-attention (*N* = 1024 tokens)
Alignment strategy	Required near-perfect pre-alignment	Multi-stage pipeline (SIFT → ORB → FFD)
Stain robustness	SSIM σ = 0.18:0.23 across protocols	SSIM σ = 0.0597 (fusion mechanism)
Training data	Paired H&E/MT required per institution	Unsupervised; no site-specific retraining
Clinical deployment	Requires per-institution recalibration	Plug-and-play integration with software like QuPath v0.6.0
Fibrosis staging accuracy	METAVIR agreement: κ = 0.68	METAVIR agreement: κ = 0.82 (projected)

**Table 2:** Summary of dataset challenges and the proposed methodological solutions in the H&E-to-MT virtual staining framework.

Challenge	Proposed Solution
Misalignment between H&E and MT slides due to use of consecutive tissue sections, leading to spatial discrepancies.	Multi-stage alignment pipeline: (i) Coarse alignment using SIFT on WSI thumbnails. (ii) Patch-level alignment via ORB + homography (RANSAC). (iii) Fine-grained local deformation correction using free-form deformation (FFD) with B-spline registration.
Minor rotational and translational misalignments introduced during slide scanning or handling.	Addressed within the same alignment pipeline: rigid-body (global) registration via SIFT/ORB, followed by non-rigid FFD for residual local shifts.
Inconsistent tissue placement on glass slides (e.g., rotation, offset, cropping differences).	Tissue region detection via contour extraction from thumbnails; patches extracted only from overlapping tissue regions using center-aligned bounding boxes.
Staining quality inconstancy across slides (e.g., over/under-staining, reagent degradation, thickness variation).	Stain normalization using Reinhard’s color transfer method in LAB color space to standardize appearance across all input images.
Artifacts such as tissue folding, air bubbles, or uneven staining.	Patch-level quality filtering: Discard patches with >30% empty regions or low similarity (MI <0.35, Cosine <0.75, pHash >20).
Computational burden from extremely large WSI dimensions (>100,000 × 100,000 pixels).	Tile-based processing: Extract 256×256 patches from aligned ROIs; process in manageable batches while preserving spatial context.

**Table 3:** Tabular presentation of the four distinct configurations utilized in this study as they were carefully designed to assess the trade-offs between identity preservation and cycle-consistency (where each configuration emphasizes specific hyperparameters and provides either original or Reinhard-normalized images to enhance the stain translation process).

Configuration	Emphasis	Hypothesis	Hyperparameters
Original with Emphasis on $\lambda_{\mathrm{identity}}$	The model was trained on the original images with a strong emphasis on the identity loss ($\lambda_{\mathrm{identity}}$)	This setup ensures that the model preserves the structural and textural integrity of the input images when no translation is required	The hyperparameter $\lambda_{\mathrm{identity}}$ was scaled by a factor of 3, while $\lambda_{\mathrm{cycle}}$ was set to its default value
Original with Emphasis on $\lambda_{\mathrm{cycle}}$	This configuration focuses on enforcing cycle-consistency in the original images	This setup ensures that the translated images maintain semantic and structural alignment between the source and target domains	The hyperparameter $\lambda_{\mathrm{cycle}}$ was scaled by a factor of 3, while $\lambda_{\mathrm{identity}}$ was set to its default value
Reinhard-Normalized with Emphasis on $\lambda_{\mathrm{identity}}$	The model was trained on Reinhard-normalized images with a strong emphasis on the identity loss ($\lambda_{\mathrm{identity}}$)	The normalization process aligns the color distribution of the input images to a target image, ensuring consistency in color appearance	The hyperparameter $\lambda_{\mathrm{identity}}$ was scaled by a factor of 3, while $\lambda_{\mathrm{cycle}}$ was set to its default value
Reinhard-Normalized with Emphasis on $\lambda_{\mathrm{cycle}}$	This configuration focuses on enforcing cycle-consistency in Reinhard-normalized images	This setup ensures that the translated images are both realistic and consistent with the original data while also benefiting from the color normalization process	The hyperparameter $\lambda_{\mathrm{cycle}}$ was scaled by a factor of 3, while $\lambda_{\mathrm{identity}}$ was set to its default value

**Table 4:** Tabular presentation of the quantitative analysis of the discussed metrics.

Metric	Equation	Category	Benefit	Insights
Mutual Information (MI)	H(X)+H(Y)−H(X,Y)	Similarity	Measures statistical dependence between images.	Evaluates global similarity and structural alignment.
Structural Similarity Index (SSIM) [53]	$\frac{\left(2\times \mu_{X}\times \mu_{Y}+C_{1}\right)\times \left(2\times \sigma_{XY}+C_{2}\right)}{\left(\mu_{X}^{2}+\mu_{Y}^{2}+C_{1}\right)\left(\sigma_{X}^{2}+\sigma_{Y}^{2}+C_{2}\right)}$	Similarity	Evaluates structural similarity.	Assesses structural alignment, luminance, and contrast.
Normalized Cross-Correlation (NCC)	$\frac{\sum \left(X-\mu_{X}\right)\times \left(Y-\mu_{Y}\right)}{\sqrt{\sum {\left(X-\mu_{X}\right)}^{2}\times \sum {\left(Y-\mu_{Y}\right)}^{2}}}$	Similarity	Measures normalized correlation.	Evaluates pixel-wise alignment and color consistency.
Cosine Similarity (CS)	$\frac{X\cdot Y}{\left|X||Y\right|}$	Similarity	Quantifies the angle between image vectors.	Assesses global similarity and texture preservation.
Histogram Intersection (HistInt)	$\sum \min(H_{X},H_{Y})$	Similarity	Measures overlap between histograms.	Evaluates color consistency and texture preservation.
Universal Quality Index (UQI) [54]	$\frac{4\times \sigma_{XY}\times \mu_{X}\times \mu_{Y}}{\left(\sigma_{X}^{2}+\sigma_{Y}^{2}\right)\times \left(\mu_{X}^{2}+\mu_{Y}^{2}\right)}$	Similarity	Evaluates image quality.	Assesses luminance, contrast, and structural alignment.
Mean Squared Error (MSE)	$\frac{1}{N}\times \sum {\left(X-Y\right)}^{2}$	Dissimilarity	Measures average squared difference.	Evaluates pixel-wise accuracy and color consistency.
Earth Mover’s Distance (EMD) [55]	$\inf_{\gamma \in \Gamma (X,Y)}\int \left|x-y\right|,d\gamma \left(x,y\right)$	Dissimilarity	Quantifies the work required to transform one image into another.	Assesses global alignment and texture preservation.
Perceptual Hash (pHash) [56]	$\mathrm{Hamming}({\mathrm{hash}}_{X},{\mathrm{hash}}_{Y})$	Dissimilarity	Measures perceptual similarity.	Evaluates perceptual quality and visual artifacts.
Jensen–Shannon Divergence (JSD) [57]	$\frac{1}{2}\times \left(\mathrm{KL}(X|M)+\mathrm{KL}(Y|M)\right)$	Dissimilarity	Quantifies the difference between probability distributions.	Assesses texture similarity and color consistency.
Blind/Referenceless Image Spatial Quality Evaluator (BRISQUE) [58]	BRISQUE(X)=Model(X)	Dissimilarity	Evaluates perceptual quality without a reference.	Evaluates perceptual quality and visual artifacts.

**Table 5:** Tabular presentation of the utilized hyperparameters.

Parameter	Value	Rationale
Input dimensions	256 × 256 × 4 (RGBA)	Balance between contextual information and GPU memory constraints
Batch size	1	Required due to memory limitations of 6GB GPU
Optimizer	Adam	Stable convergence for GAN training
Learning rate	$10\times 10^{-5}$	Prevents mode collapse while ensuring convergence
$beta_{1}$, $beta_{2}$	0.5, 0.999	Standard for GAN stability
Training epochs	200	Determined via early stopping (val loss plateau)
$\lambda_{cycle}$	10 (default), 3 (emphasized)	Enforces cycle-consistency
$\lambda_{identity}$	10 (default), 3 (emphasized)	Preserves structural identity
Patch overlap	32 pixels	Maintains spatial context during tile extraction
Training/validation split	27 WSIs: 80%/20% (patient-level)	Prevents data leakage across subjects
Device	Windows 11/6 GB GPU/256 GB RAM	GPU of optimization and memory for data

**Table 6:** Quantitative analysis of the proposed approach across 24 trials and four approaches alongside the fusion between them. The performance metrics for the testing subset (including MI, SSIM, and GMSD) are presented. The mean ± standard deviation values are reported to demonstrate the consistency and reliability of the proposed approach.

Metric	O/10/3	O/3/10	R/10/3	R/3/10	Fused
MI (↑)	0.9145 ± 0.084	0.946 ± 0.0918	0.8883 ± 0.0822	0.9183 ± 0.0891	0.9815 ± 0.0934
NMI (↑)	0.2561 ± 0.0169	0.2639 ± 0.0182	0.252 ± 0.0163	0.2572 ± 0.0172	0.2678 ± 0.0184
SSIM (↑)	0.7137 ± 0.0576	0.735 ± 0.0567	0.7102 ± 0.0621	0.7233 ± 0.062	0.7474 ± 0.0597
NCC (↑)	0.9209 ± 0.0253	0.9257 ± 0.0256	0.9179 ± 0.0238	0.9225 ± 0.0235	0.932 ± 0.022
CS (↑)	0.9939 ± 0.0013	0.9943 ± 0.0014	0.9931 ± 0.0018	0.9935 ± 0.002	0.9946 ± 0.0014
HistInt (↑)	0.8917 ± 0.057	0.891 ± 0.0606	0.8538 ± 0.0685	0.8528 ± 0.0761	0.8733 ± 0.0685
PSNR (↑)	30.0217 ± 0.2314	30.0954 ± 0.2629	29.8645 ± 0.2464	29.9013 ± 0.2849	30.1037 ± 0.3247
FBS (↑)	0.1874 ± 0.0572	0.2192 ± 0.0628	0.1745 ± 0.0509	0.201 ± 0.0584	0.2338 ± 0.0651
UQI (↑)	0.9183 ± 0.0266	0.9229 ± 0.0272	0.9128 ± 0.029	0.9166 ± 0.0294	0.9286 ± 0.0248
SRS (↑)	0.9333 ± 0.0215	0.9373 ± 0.0219	0.9278 ± 0.0219	0.9325 ± 0.0217	0.9415 ± 0.0198
PC (↑)	0.9028 ± 0.0325	0.9087 ± 0.0319	0.8971 ± 0.0336	0.9022 ± 0.0334	0.9134 ± 0.0302
NQM (↑)	7.4164 ± 0.4671	7.3047 ± 0.4274	7.8393 ± 0.8897	7.847 ± 1.0481	7.4815 ± 0.763
MSE (↓)	64.8824 ± 3.4925	63.8301 ± 3.9572	67.318 ± 3.8438	66.8074 ± 4.4377	63.8018 ± 4.8184
NMSE (↓)	0.1581 ± 0.0506	0.1485 ± 0.0512	0.1642 ± 0.0475	0.155 ± 0.0471	0.1359 ± 0.0439
EMD (↓)	4.3745 ± 3.1701	4.3383 ± 3.2906	6.9242 ± 4.2476	7.0563 ± 4.8512	5.1765 ± 3.739
HD (↓)	0.0849 ± 0.0304	0.0859 ± 0.033	0.1097 ± 0.0396	0.1112 ± 0.0465	0.0897 ± 0.0419
BhD (↓)	0.0087 ± 0.0076	0.0091 ± 0.0087	0.0157 ± 0.0133	0.0165 ± 0.016	0.011 ± 0.0109
pHash (↓)	6.8566 ± 1.5391	6.5555 ± 1.4951	7.0065 ± 1.4431	6.9102 ± 1.4239	6.3476 ± 1.3693
JSD (↓)	0.044 ± 0.0051	0.0426 ± 0.0055	0.0458 ± 0.0058	0.0446 ± 0.0062	0.0408 ± 0.0052
KLD (↓)	0.0335 ± 0.0368	0.0349 ± 0.0407	0.0593 ± 0.0504	0.0649 ± 0.0636	0.0441 ± 0.0427
BRISQUE (↓)	15.5911 ± 3.0334	15.5911 ± 3.0334	15.5911 ± 3.0334	15.5911 ± 3.0334	15.2632 ± 5.958
GMSD (↓)	0.206 ± 0.0096	0.2023 ± 0.01	0.2082 ± 0.0103	0.2053 ± 0.0106	0.2009 ± 0.0105

**Table 7:** ANOVA results and Tukey HSD pairwise comparisons with 95% confidence intervals for key similarity metrics. All *p*-values are Bonferroni-adjusted. Confidence intervals represent mean difference (fused − configuration).

Metric	F-Statistic	*p*-Value	Fused vs. O/10/3	Fused vs. O/3/10	Fused vs. R/10/3	Fused vs. R/3/10
MI	190.89	<0.001	[0.057, 0.077]	[0.028, 0.043]	[0.082, 0.105]	[0.056, 0.070]
NMI	124.41	<0.001	[0.009, 0.014]	[0.002, 0.006]	[0.013, 0.018]	[0.009, 0.012]
SSIM	138.46	<0.001	[0.027, 0.040]	[0.008, 0.016]	[0.034, 0.040]	[0.021, 0.027]
NCC	45.92	<0.001	[0.008, 0.014]	[0.003, 0.009]	[0.012, 0.017]	[0.008, 0.011]
CS	17.07	<0.001	[0.000, 0.001]	[0.000, 0.001]	[0.001, 0.002]	[0.001, 0.002]

**Table 8:** Effect sizes (Cohen’s *d*) for fused versus individual configurations and median improvement ranges (fused vs. individual configs). Values >0.8 indicate large effects.

Metric	O/10/3	O/3/10	R/10/3	R/3/10
MI	4.03	2.93	4.93	5.58
NMI	2.93	1.27	4.03	4.39
SSIM	3.10	1.78	7.17	5.28
NCC	2.00	1.21	3.30	3.53
CS	1.11	0.77	2.02	1.19
**Median improvement range (fused vs. individual configs)**				
MI	2.87%:9.73%			
NMI	2.00%:7.62%			
SSIM	1.79%:5.14%			
NCC	0.56%:1.43%			
CS	0.02%:0.15%			

**Table 9:** Empirically validated model complexity metrics for the TbGAN framework under actual training conditions (NVIDIA RTX A2000 12 GB VRAM). All values measured during 50-epoch training on 27 WSIs with 256 × 256 × 4 (RGBA) inputs.

Metric	Value	Clinical Relevance
Total parameters	31.3 million	Fits within memory constraints of mid-tier clinical GPUs
(a) Generator (transformer)	28.5 million	Captures long-range collagen dependencies
(b) Discriminator (CNN)	2.8 million	Efficient patch-level realism assessment
Training time (50 epochs)	68.3 h	Feasible for offline model development
Inference speed per tile	1.8 s	Real-time capable for diagnostic workflows
Peak training memory	1253 MB	20.9% of 6 GB GPU capacity
Activation memory (inference)	289 MB	Minimal overhead during deployment
Sequence length (tokens)	1024	Derived from 8 × 8 patch embedding kernel
Attention matrix memory	384 MB	Dominant memory consumer due to $O(N^{2})$ scaling
Gradient accumulation steps	8	Simulates effective batch size of 8 to stabilize optimization

## Data Availability

The original contributions presented in the study are included in the article, further inquiries can be directed to the corresponding author.
